# Supplementary material for: Unifying phylogenetic traversal and deep learning to guide tree exploration
Source: bioRxiv. 2026 Jan 14:2026.01.14.699358. Preprint. [Version 1] doi: 10.64898/2026.01.14.699358 (PMC12871347; doi:10.64898/2026.01.14.699358)
Supplement: 1 [file NIHPP2026.01.14.699358V1-supplement-1.pdf]

bioRxiv preprint doi: https://doi.org/10.64898/2026.01.14.699358; this version posted January 14, 2026. The copyright holder for this preprint (which was not certified by peer review) is the author/funder, who has granted bioRxiv a license to display the preprint in perpetuity. It is made available under aCC-BY 4.0 International license.

Supplementary Materials

Supplementary figures

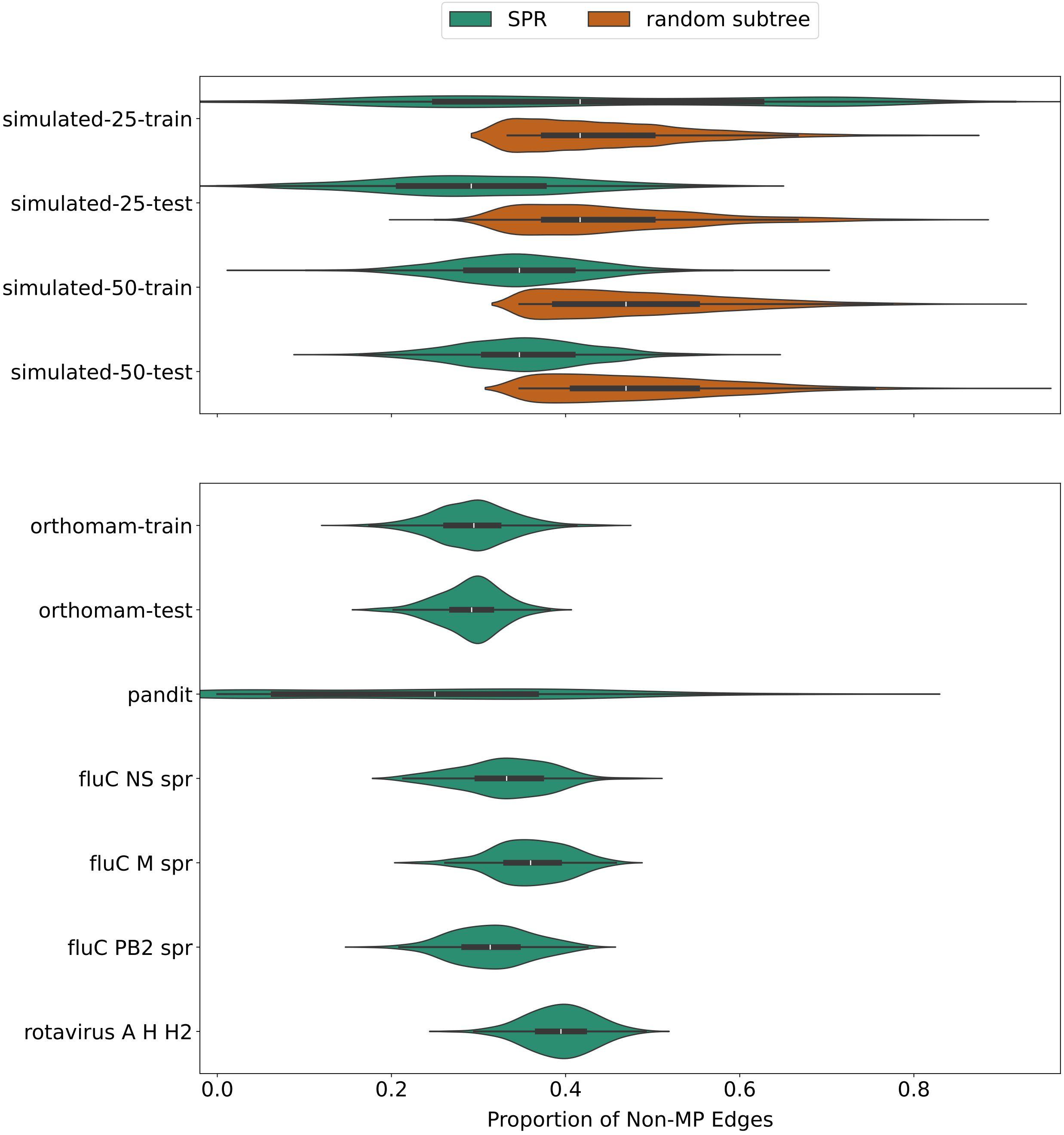

Figure S1: Fraction of non-MP edges in simulated (top) and empirical (bottom) datasets presented in this paper. For simulated datasets we distinguish the different perturbation methods used to introduce non-MP edges (“SPR” vs “random subtree”)

bioRxiv preprint doi: https://doi.org/10.64898/2026.01.14.699358; this version posted January 14, 2026. The copyright holder for this preprint (which was not certified by peer review) is the author/funder, who has granted bioRxiv a license to display the preprint in perpetuity. It is made available under aCC-BY 4.0 International license.

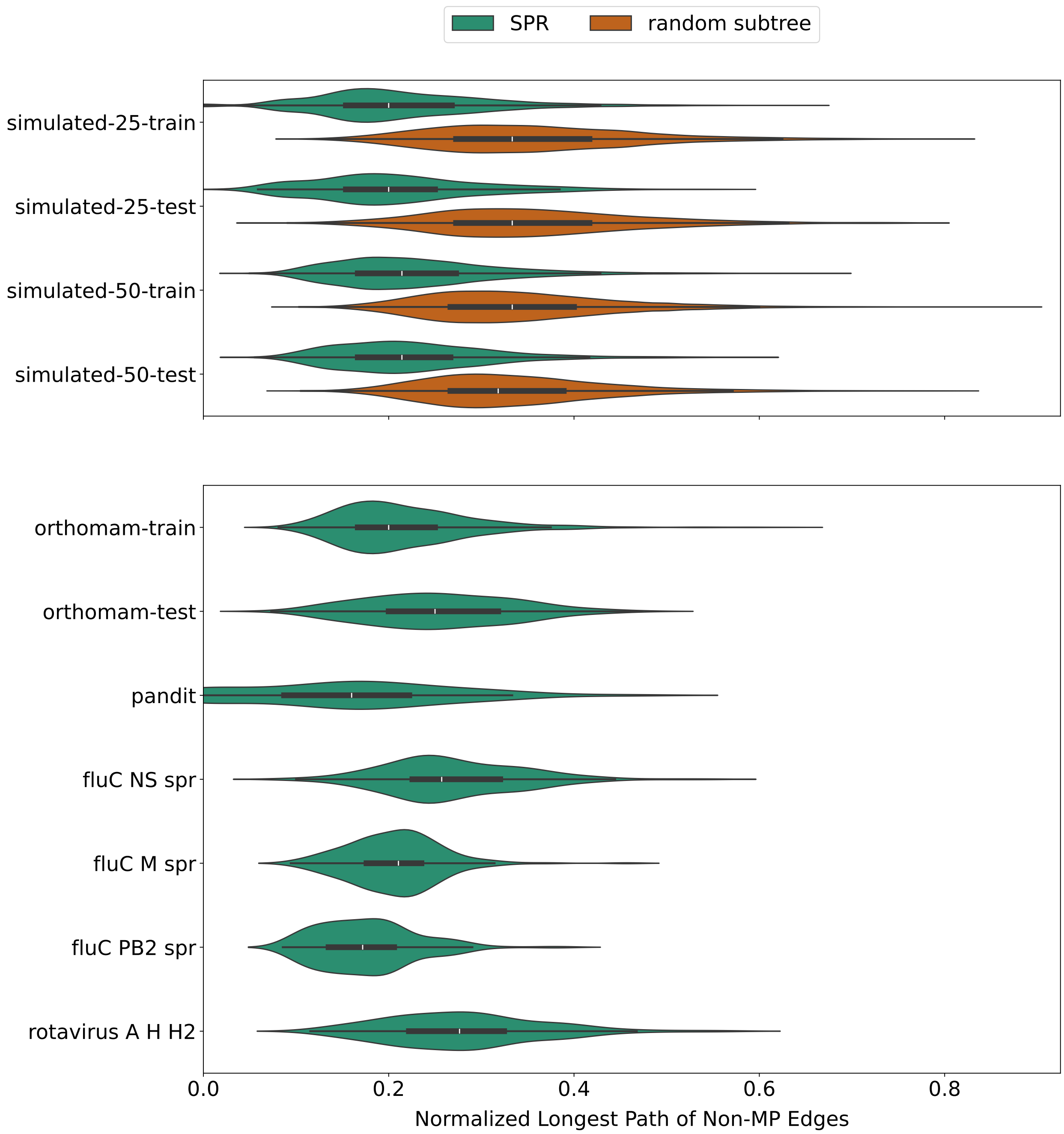

Figure S2: Normalized length of longest non-MP path in simulated (top) and empirical (bottom) datasets presented in this paper. For every tree in the dataset, we take the longest directed path in the tree, i.e. a path is required to be a sub-path of a path from the root to a leaf. For simulated datasets we distinguish the different perturbation methods used to introduce non-MP edges ("SPR" vs "random subtree")

bioRxiv preprint doi: https://doi.org/10.64898/2026.01.14.699358; this version posted January 14, 2026. The copyright holder for this preprint (which was not certified by peer review) is the author/funder, who has granted bioRxiv a license to display the preprint in perpetuity. It is made available under aCC-BY 4.0 International license.

Complexity of the MP Edge Problem

Problem 1 (MP Edge Problem)**.** *Let A be an alignment, $S_1|S_2$ a split of A, and k an integer. Is there a tree T for A containing an edge inducing the split $S_1|S_2$ with $\mathrm{ps}_A(T) \leq k$?*

Theorem 1. *The* MP Edge Problem *is NP-complete.*

We prove Theorem 1 by reducing the *NP*-complete problem (Day and Sankoff, 1986) of finding a maximum parsimony tree (MP Problem) to the MP Edge Problem.

Problem S1 (MP Problem)**.** *Let A be an alignment and k an integer.*

Is there a tree T for A that has parsimony score $\mathrm{ps}_A(T) \leq k$?

Proof of Theorem 1. Given a solution to MP Edge Problem, i.e. a tree T for alignment A that contains an edge inducing the split $S_1|S_2$, we can use the Fitch algorithm to compute its parsimony score and verify whether it is less than or equal to the given integer k. Therefore, MP Edge Problem is in the class *NP*.

To show *NP*-hardness, we reduce MP Problem to MP Edge Problem. Let A be an alignment containing sequences $s_1, s_2, \ldots, s_n$ and k be an integer that give us an instance of MP Problem. We use $A' = A$, $l = k$, and the split $S = \{s_1\}|\{s_2, s_3, \ldots, s_n\}$ as an instance of MP Edge Problem. We now need to prove that there is a tree T for A with $\mathrm{ps}_A(T) \leq k$ if and only if there is a tree T' for A' containing an edge inducing the split S with $\mathrm{ps}_{A'}(T') \leq l$. With $A' = A$, $l = k$, and S being a trivial split that is present in any tree for A, it is easy to see that this is true with $T = T'$. □
